# Combined impact of elevated C-reactive protein levels and dyslipidemia on stroke: a CHNS prospective cohort study

**DOI:** 10.3389/fpubh.2024.1435004

**Published:** 2024-08-23

**Authors:** Meiru Lv, Chang Su, Feifei Huang, Xiaofang Jia, Jiguo Zhang, Huijun Wang, Xiaoyu Wu, Weiwei Zhou, Wenwen Du

**Affiliations:** ^1^National Institute for Nutrition and Health, Chinese Center for Disease Control and Prevention, Beijing, China; ^2^Key Laboratory of Trace Element Nutrition of Health Commission of China, Beijing, China

**Keywords:** C-reactive protein, dyslipidemia, stroke, interaction, health

## Abstract

**Background:**

The objective of this study was to examine whether the combination of elevated levels of C-reactive protein (CRP) and dyslipidemia increased the risk of stroke among middle-aged and older adult individuals in China.

**Methods:**

This study utilized longitudinal data from the China Health and Nutrition Survey (CHNS) collected in 2009, 2015, and 2018. A total of 8,023 participants aged ≥40 years (3,595 males and 4,428 females) were included. The Generalized Estimating Equation (GEE) method was employed to examine the association between inflammation, dyslipidemia, their combined effects, and stroke in the Chinese population.

**Results:**

A total of 174 stroke events occurred during follow-up. Compared with those with normal CRP levels (CRP ≤ 3 mg/L), the adjusted ORs and 95%CI were 2.13 (1.25, 3.64) for the female with elevated CRP level. Compared with those with non-dyslipidemia, the adjusted ORs and 95%CI were 1.56 (1.03, 2.37) for the individuals with high LDL cholesterol, 1.93 (1.12, 3.33) for the male with high LDL cholesterol. Compared with those with normal CRP levels and non-dyslipidemia, the adjusted ORs and 95%CI were 1.74 (1.08, 2.78) for the individuals with elevated CRP levels and dyslipidemia, 2.41 (1.29, 4.49) for the male with elevated CRP levels and dyslipidemia. People with the coexistence of elevated CRP levels and dyslipidemia had the highest risk of stroke among male.

**Conclusion:**

In females, higher levels of inflammation are associated with an increased incidence of stroke. In males, individuals with dyslipidemia characterized by high LDL cholesterol levels are more susceptible to stroke. In the general population, the joint effect of inflammation and dyslipidemia predisposes individuals to a higher risk of stroke, particularly among males.

## 1 Introduction

Stroke is the second-leading cause of disability and death worldwide, with the highest burden of the disease shared by low- and middle-income countries (1). The number of new stroke cases in China in 2019 was 3.94 million, with an incidence rate of 276.7 (241.3 to 322.0) per 100,000 people, an increase of 86.0% compared to 1990 (2). According to survey data from China, the incidence rate of stroke has shown a continuous upward trend in most provinces over the past seven years (2013–2019). This increase in stroke cases is particularly notable among males, older individuals, and those living in rural areas (3).

C-reactive protein (CRP) is an acute-phase reactant that is produced by hepatocytes, which are liver cells. It is considered a biomarker of inflammation because its levels in the blood increase in response to inflammation or tissue damage in the body. High-sensitivity CRP (hs-CRP) assays are specifically designed to accurately measure low levels of CRP in the blood (4). The high-sensitivity C-reactive protein (hs-CRP) level is a measure of the body's inflammatory response to atherosclerosis and serves as a peripheral marker of inflammation (5). It has been found that complications associated with vulnerable atherosclerotic plaques globally are mainly triggered by dyslipidemia and inflammatory mechanisms (6). The effects of inflammation and dyslipidemia on stroke have not yet been studied in depth in the Chinese population. The aim of this paper is to explore the impact of C-reactive protein, dyslipidemia and the combination of the two on the development of stroke.

## 2 Methods

### 2.1 Study population

This study utilized data from three waves of the China Health and Nutrition Survey (CHNS) conducted in 2009, 2015, and 2018 to investigate the relationship between inflammation, dyslipidemia, and stroke. The CHNS project started in 1989 and expanded its survey coverage to nine provinces in China including Heilongjiang, Liaoning, Shandong, Henan, Hunan, Hubei, Guangxi, Guizhou, and Jiangsu by 2009. In 2011, it included Beijing, Shanghai, and Chongqing, and in 2015, Zhejiang, Shanxi, and Yunnan provinces were added. The survey employed a multi-stage, cluster random sampling method to collect information on communities, households, individuals, diet, and biological factors. Dietary information was collected using a 3-day 24-h dietary recall, household food weighing method, and food frequency questionnaire (7, 8).

**Figure 1:**
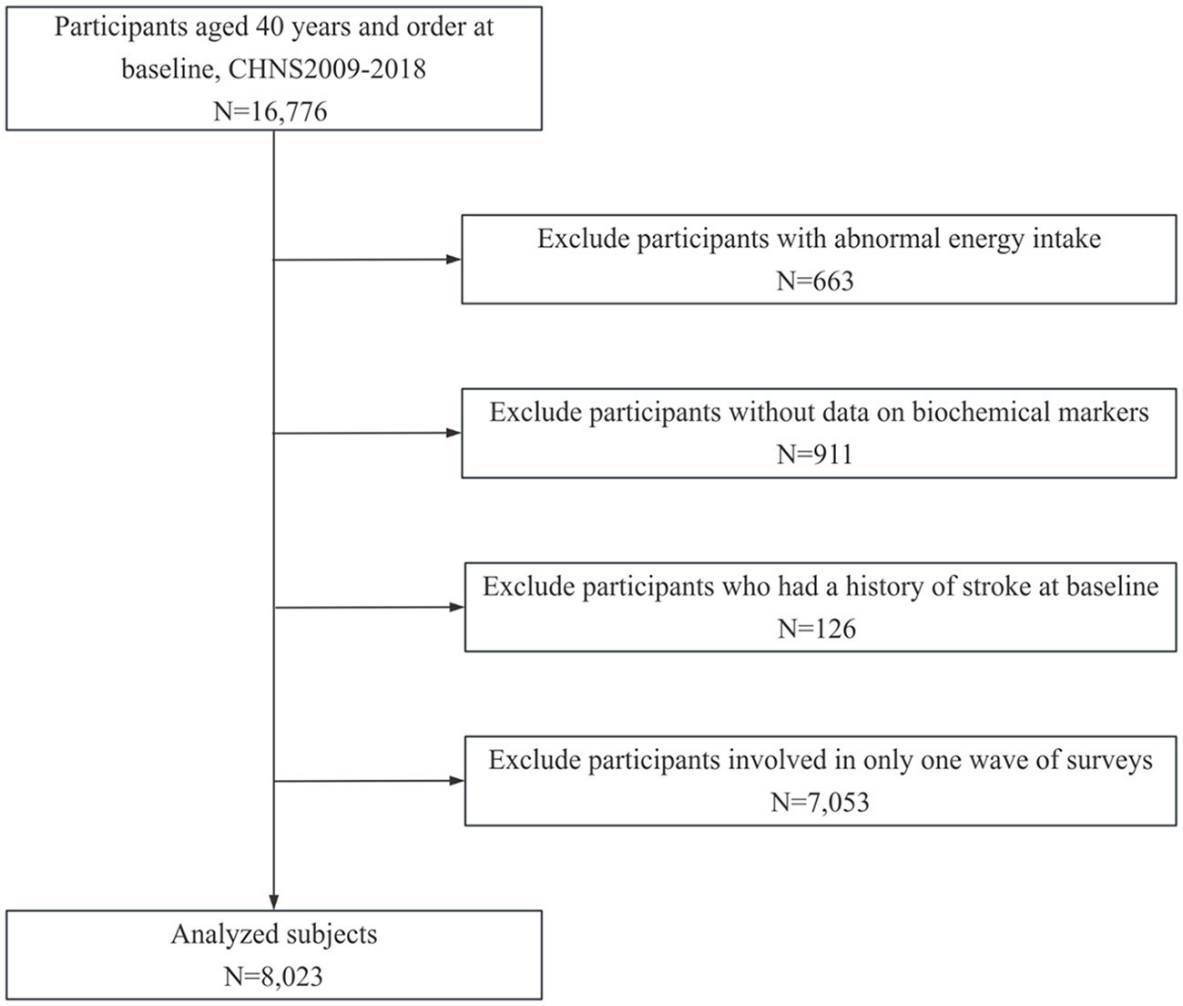
Flowchart for the selection of analyzed cohort.

The CHNS project data from 2009, 2015, and 2018 were used in this study, encompassing a total of 16,776 individuals. Initially, 663 individuals with abnormal energy intake were excluded. Abnormal energy intake was defined as daily energy intake < 500 kcal or >5,000 kcal. Furthermore, 911 individuals with missing biochemical markers were excluded. Additionally, 126 individuals with pre-existing conditions at baseline were also excluded. Lastly, 7,053 individuals who had less than two follow-up rounds were excluded. The final sample for this study consisted of 8,023 individuals (Figure 1). We divided the study participants into four groups: normal CRP levels and non-dyslipidemia (8,818), elevated CRP levels and non-dyslipidemia (1,320), normal CRP levels and dyslipidemia (4,813), and elevated CRP levels and dyslipidemia (1,095). This grouping was done to analyze the combined effect of elevated inflammatory levels and dyslipidemia on the risk of stroke.

### 2.2 Assessment of critical variables

In this subject, the inflammatory marker is C-reactive protein, with a normal range of less than or equal to three. If it exceeds three, it is considered to indicate inflammation (9).

Biochemical tests for blood lipid include triglyceride (TG), total cholesterol (TC), low-density lipoprotein cholesterol (LDL-C), and high-density lipoprotein cholesterol (HDL-C). The diagnostic criteria are ≥2.3 mmol/L, ≥6.2 mmol/L, ≥4.1 mmol/L, and < 1.0 mmol/L, respectively. If any one or more of these criteria are met, it can be diagnosed as dyslipidemia (10).

In this study, stroke is defined according to the specific question, “Has a doctor ever given you the diagnosis of stroke or transient ischemic attack?”

### 2.3 Assessment of covariates

Information on socio-demographic and lifestyle variables were collected through standard questionnaires by trained interviewers, including age (in years); sex (male/female); education level (primary school/middle school/high school and above); geographic region (urban and rural); marital status (married/other); average household income (RMB/year); smoking (yes/no); drinking (yes/no); BMI (< 18.5/18.5~23.9/24.0~27.9/≥28.0); physical activity (in MET hours/week); hypertension (yes/no), diabetes (yes/no), tumor (yes/no). In addition, we also assessed other potential dietary confounders, including total energy intake, intakes of dietary fat, cholesterol, vegetable, sodium, and red meat calculated from data collected by consecutive 3 days 24 h recalls combined with the weighing of household seasonings.

### 2.4 Biological detection methods

All samples were analyzed at a nationally accredited central laboratory in Beijing. The laboratory has obtained ISO 15189:2007 certification for medical laboratories and strictly adheres to quality control measures. HbA1c levels were evaluated using a high-performance liquid chromatography system manufactured by Tosoh Corporation in Tokyo, Japan. The assessment of dyslipidemia markers, including total cholesterol (TC), high-density lipoprotein cholesterol (HDL-C), low-density lipoprotein cholesterol (LDL-C), and triglycerides (TG), was carried out using the glycerol-phosphate oxidase method and the polyethylene glycol (PEG)-modified enzyme method. The reagents from Kyowa Medex Co., Ltd in Tokyo, Japan, were used for this purpose. All lipid measurements were performed on the Hitachi 7600 automated analyzer from Hitachi Inc. in Tokyo, Japan. The assessment of inflammatory status, indicated by high sensitivity C-reactive protein (CRP) levels, was conducted using the immunoturbidimetric method with Denka Seiken reagents, also sourced from Japan. This analysis was carried out on the Hitachi 7600 automated analyzer (11).

### 2.5 Statistical analysis

In this study, continuous variables were described using mean and standard deviation, while categorical variables were described using chi-square tests, rank-sum tests, and other appropriate methods. The associations between inflammation, blood lipid abnormalities, and stroke were explored using Generalized Estimate Equation (GEE). We constructed four sequential models for analysis: Model 1 adjusted for age, sex, education level, geographic region, marital status, and average household income; Model 2 further adjusted for smoking, drinking, BMI, and physical activity; Model 3 further adjusted for dietary fat, cholesterol, vegetable, energy, sodium, and red meat; Model 3 further adjusted for hypertension, dyslipidemia, diabetes, and tumor. All the aforementioned analyses were implemented in R.

## 3 Results

This study utilized data from the China Health and Nutrition Survey, including a total of 8,023 participants, which consisted of 3,595 males and 4,428 females. This study investigated the combined effects of inflammation and blood lipid abnormalities on stroke. The study subjects were divided into four groups: Normal CRP levels and non-dyslipidemia (4,393), Elevated CRP levels and non-dyslipidemia (660), Normal CRP levels and dyslipidemia (2,416), Elevated CRP levels, and dyslipidemia (554). The average age of the study participants was 58.7 ± 10.6. In the population with elevated inflammatory levels and abnormal lipid profiles, it is commonly observed that they are predominantly married women with lower levels of education, residing in rural areas, unemployed, and have insufficient physical activity levels. They also tend to have a higher prevalence of overweight and obesity. However, the proportion of individuals who smoke or consume alcohol is relatively low, which may be associated with gender differences (Table 1).

**Table 1:** Baseline characters of participants grouped by elevated CRP levels and dyslipidemia.

**Variables (%)**	**Total**	**Normal CRP levels and non-dyslipidemia**	**Elevated CRP levels and non-dyslipidemia**	**Normal CRP levels and dyslipidemia**	**Elevated CRP levels and dyslipidemia**
Age (years)^***^	55.9 ± 10.1	55.3 ± 10.1	58.9 ± 10.7	55.5 ± 9.6	58.2 ± 10.2
**Gender, (%)** ^***^					
Male	3,595 (44.8)	1,877 (42.7)	292 (44.2)	1,202 (49.8)	224 (40.4)
Female	4,428 (55.2)	2,516 (57.3)	368 (55.8)	1,214 (50.2)	330 (59.6)
**Marital status, (%)**					
Married	7,460 (93.0)	4,101 (93.4)	601 (91.1)	2,241 (92.8)	517 (93.3)
Other	563 (7.0)	292 (6.7)	59 (8.9)	175 (7.2)	37 (6.7)
**Education level, (%)** ^***^					
Primary school	3,519 (43.9)	1,968 (44.8)	324 (49.1)	970 (40.1)	257 (46.4)
Middle school	2,483 (30.9)	1,366 (31.1)	186 (28.2)	772 (32.0)	159 (28.7)
High school and above	2,021 (25.2)	1,059 (24.1)	150 (22.7)	674 (27.9)	138 (24.9)
**Geographic region, (%)** ^***^					
Urban	2,763 (34.4)	1,415 (32.2)	245 (37.1)	893 (37.0)	210 (37.9)
Rural	5,260 (65.6)	2,978 (67.8)	415 (62.9)	1,523 (63.0)	344 (62.1)
**Working status, (%)** ^***^					
Yes	4,066 (50.7)	2,350 (53.5)	313 (47.4)	1,173 (48.6)	230 (41.5)
No	3,957 (49.3)	2,043 (46.5)	347 (52.6)	1,243 (51.4)	324 (58.5)
**Smoking, (%)** ^**^					
Yes	2,305 (28.7)	1,213 (27.6)	181 (27.4)	760 (31.5)	151 (27.3)
No	5,718 (71.3)	3,180 (72.4)	479 (72.6)	1,656 (68.5)	403 (72.7)
**Drinking, (%)** ^*^					
Yes	2,420 (30.2)	1,289 (29.3)	185 (28.0)	784 (32.5)	162 (29.2)
No	5,603 (69.8)	3,104 (70.7)	475 (72.0)	1,632 (67.5)	392 (70.8)
**BMI (kg/m** ^2^ **), (%)** ^***^					
< 18.5	277 (3.5)	199 (4.5)	42 (6.4)	33 (1.4)	3 (0.5)
18.5~23.9	3,854 (48.0)	2,484 (56.5)	281 (42.6)	932 (38.6)	157 (28.3)
24.0~27.9	2,886 (36.0)	1,371 (31.2)	213 (32.3)	1,058 (43.8)	244 (44.0)
≥28.0	1,006 (12.5)	339 (7.7)	124 (18.8)	393 (16.3)	150 (27.1)
Physical activity (met·hours/week), (%)^***^	185.7 ± 195.1	202 ± 201	180 ± 188	166 ± 185	153 ± 187
Average household income (RMB/year)	16,377 ± 30,261.3	16,109 ± 34,327	14,249 ± 16,112	17,337 ± 25,527	16,846 ± 27,576

^***^P < 0.0001; ^**^P < 0.01; ^*^P < 0.05.

In the study of the relationship between CRP levels and stroke, it was found that higher CRP levels in Chinese women are associated with an increased risk of stroke, with a risk 2.13 times higher than that of the normal population. However, no significant association was found between CRP levels and stroke risk in the overall population or among males (Table 2).

**Table 2:** Odd ratios (OR) and 95% confidence intervals (95% CI) of stroke according to serum hs-CRP concentrations.

	**CRP (mg/L)**	
	**Normal CRP levels**	**Elevated-CRP levels**
**Total**		
Number of participants	13,631	2,415
Number of stoke cases	127	47
**OR (95%CI)**		
Model 1	1	1.64 (1.13, 2.36)
Model 2	1	1.55 (1.07, 2.25)
Model 3	1	1.56 (1.08, 2.25)
Model 4	1	1.40 (0.97, 2.03)
**Males**		
Number of participants	6,132	1,058
Number of cases	85	23
**OR (95%CI)**		
Model 1	1	1.19 (0.71, 1.99)
Model 2	1	1.22 (0.72, 2.05)
Model 3	1	1.21 (0.72, 2.03)
Model 4	1	1.06 (0.62, 1.79)
**Females**		
Number of participants	7,499	1,357
Number of cases	42	24
**OR (95%CI)**		
Model 1	1	2.60 (1.54, 4.37)
Model 2	1	2.24 (1.31, 3.81)
Model 3	1	2.27 (1.33, 3.89)
Model 4	1	2.13 (1.25, 3.64)

Model 1: age, sex, education level, geographic region, marital status, and average household income. Model 2: model 1 with smoking, drinking, BMI, and physical activity. Model 3: model 2 with dietary fat, cholesterol, vegetable, energy, sodium, and red meat. Model 4: model 3 with hypertension, dyslipidemia, diabetes, and tumor.

The study of the association between dyslipidemia and stroke risk found no significant association between dyslipidemia and stroke incidence in the overall population. After stratifying the population by gender, the association remained insignificant (Supplementary material). Based on this, it was inferred that dyslipidemia may not be an independent risk factor for stroke. Dyslipidemia was further classified into four types, including high cholesterol, high triglycerides, abnormal low-density lipoprotein cholesterol levels, and abnormal high-density lipoprotein cholesterol levels. From the perspective of the overall population, after adjusting for confounding factors, it was found that low LDL cholesterol levels were positively correlated with the risk of stroke, which was also observed in males. However, no significant association was found in females for all other categories of dyslipidemia (Supplementary material).

**Figure 2:**
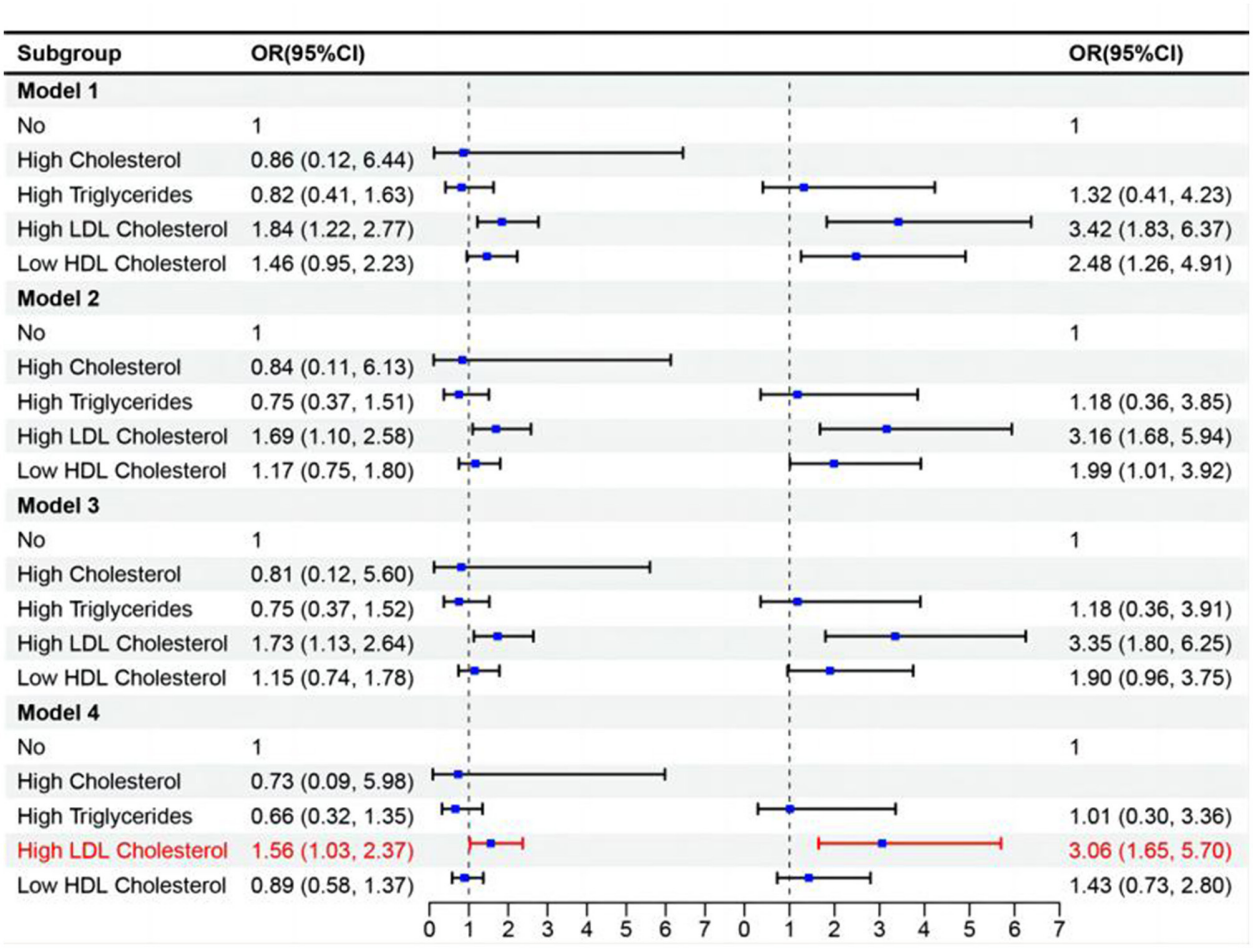
Analysis of the combined effect of different types of dyslipidemia and high levels of CRP on stroke.

The study population was further divided into four groups based on normal or abnormal C-reactive protein levels and whether they had dyslipidemia. Among the group with abnormal C-reactive protein and dyslipidemia, after adjusting for confounding factors, it was found that the risk of stroke in this group was 1.74 times higher than that of the normal population. In females, the risk of stroke was found to be 2.41 times higher in the group with abnormal C-reactive protein and dyslipidemia compared to the normal population. However, no such association was found in males (Table 3). Analysis of different types of dyslipidemia in combination with abnormal inflammation levels revealed that high LDL cholesterol combined with high levels of CRP are a risk factor for the development of stroke (Figure 2).

**Table 3:** The joint association of elevated CRP levels and dyslipidemia with stroke.

	**Normal CRP levels and non-dyslipidemia**	**Elevated CRP levels and non-dyslipidemia**	**Normal CRP levels and dyslipidemia**	**Elevated CRP levels and dyslipidemia**
**Total**				
Number of participants	8,818	1,320	4,813	1,095
Number of cases	79	19	48	28
**OR (95%CI)**				
Model 1	1	1.18 (0.70, 1.99)	1.23 (0.86, 1.77)	2.59 (1.63, 4.11)
Model 2	1	1.14 (0.67, 1.94)	1.08 (0.74, 1.58)	2.19 (1.36, 3.53)
Model 3	1	1.15 (0.68, 1.96)	1.09 (0.75, 1.59)	2.19 (1.36, 3.52)
Model 4	1	1.07 (0.62, 1.83)	0.95 (0.65, 1.38)	1.74 (1.08, 2.78)
**Males**				
Number of participants	3,794	591	2,338	467
Number of cases	52	10	33	13
**OR (95%CI)**				
Model 1	1	0.90 (0.44, 1.83)	1.29 (0.83, 2.01)	1.95 (0.98, 3.86)
Model 2	1	0.93 (0.45, 1.92)	1.16 (0.72, 1.85)	1.78 (0.88, 3.59)
Model 3	1	0.92 (0.45, 1.89)	1.17 (0.73, 1.87)	1.79 (0.89, 3.60)
Model 4	1	0.83 (0.39, 1.75)	1.00 (0.62, 1.60)	1.35 (0.66, 2.74)
**Females**				
Number of participants	5,024	729	2,475	628
Number of cases	27	9	15	15
**OR (95%CI)**				
Model 1	1	1.86 (0.87, 3.97)	1.08 (0.58, 2.04)	3.60 (1.93, 6.73)
Model 2	1	1.63 (0.74, 3.59)	0.95 (0.49, 1.81)	2.77 (1.45, 5.32)
Model 3	1	1.71 (0.77, 3.83)	0.98 (0.51, 1.89)	2.80 (1.46, 5.40)
Model 4	1	1.69 (0.76, 3.78)	0.90 (0.47, 1.75)	2.41 (1.29, 4.49)

Model 1: age, sex, education level, geographic region, marital status, and average household income. Model 2: model 1 with smoking, drinking, BMI, and physical activity. Model 3: model 2 with dietary fat, cholesterol, vegetables, energy, sodium, and red meat. Model 4: model 3 with hypertension, diabetes, and tumor.

## 4 Discussion

In this study, we investigated the association between inflammation levels and stroke in adults aged 40 and older in China. We also examined the association between dyslipidemia and stroke, as well as the combined effect of inflammation and different types of dyslipidemia on stroke. The results showed that higher levels of inflammation (>3) were positively correlated with stroke in women aged 40 and over in China. Compared to women with normal inflammation levels, women with higher inflammation levels had a 2.60-fold higher probability of stroke (OR = 2.60, 95% CI: 1.54–4.37). After adjusting for confounding factors, the probability of stroke in women with higher levels of inflammation was 2.13 times higher (OR = 2.13, 95%CI: 1.25–3.64). In a study of 90,517 Chinese adults who had no stroke or myocardial infarction at baseline, they found that people with high levels of C-reactive protein had a 1.25 times higher risk of having a stroke than the normal population. The risk was 1.25 times higher for men and for people with hypertension (12). The European prospective investigation into cancer and nutrition (EPIC)-Norfolk cohort-based study included 18,450 healthy individuals at baseline, and the results showed that CRP was associated with the occurrence of a stroke (13). In studies in Germany and the UK, CRP has been shown to be an important way to predict heart disease, like myocardial infarction and stroke (14–17). In the present study, the above associations were not found in the whole population or in men, and it is hypothesized that they may be related to the unique physiological status of women.

No association was found between dyslipidemia and stroke in the general population. However, when looking at different types of dyslipidemia, there was an association between high LDL-C dyslipidemia and stroke. Compared to the population without dyslipidemia, the probability of stroke was 1.56 times higher in individuals with high LDL-C dyslipidemia (OR = 1.56, 95% CI: 1.03–2.37). Studies both at home and abroad have shown that dyslipidemia is a risk factor for stroke (18–21). In a cross-sectional study conducted in China in 2015, 16,892 study participants were included, and after adjusting for confounders, it was found that hypertension, dyslipidemia, and physical inactivity were risk factors for stroke among adults aged 40 years and older in southwest China (22). The Chinese study showed that among people aged 40 years and older, low HDL-C dyslipidemia was predominantly found in urban areas, while other types of dyslipidemia were predominantly found in rural areas and were more common in women (23). The association between high LDL-C dyslipidemia and stroke observed in this study may be related to the distribution of different types of dyslipidemia in different populations.

Based on above studies, we conducted our study to assess the combined effect of elevated-CRP levels and dyslipidemia on the overall risk of stroke in the middle-aged and geriatric Chinese population. This study demonstrated a positive correlation between the combined effect of high levels of inflammation and dyslipidemia and stroke in the overall population. The probability of stroke was 1.74 times higher in individuals with high levels of inflammation and dyslipidemia compared to those without abnormalities (OR = 1.74, 95%CI: 1.08–2.78). In women, the probability of stroke was 2.41 times higher in those with high levels of inflammation and dyslipidemia compared to normal women (OR = 2.41, 95%CI: 1.29–4.49). Atherosclerosis is a major cause of cardiovascular disease, and dyslipidemia can be involved in the development of atherosclerosis by activating pro-inflammatory cytokines (24–27). During the development of atherosclerosis, inflammation within the vessel wall occurs due to vascular endothelial dysfunction and poor vascular smooth muscle cell plasticity (28), which in turn promotes stroke occurrence.

The combined effect of high inflammation levels and high LDL cholesterol dyslipidemia was also positively correlated with stroke in the overall population. The probability of stroke was 3.06 times higher in individuals with high inflammation levels and high LDL cholesterol dyslipidemia compared to the population without abnormalities (OR = 3.06, 95%CI: 1.65–5.70). It has been shown that in the development of atherosclerosis, LDL cholesterol can interact with vascular-related cells (endothelial, smooth muscle cells, and macrophages), which leads to the secretion of growth factors and adhesion molecules and the formation of foam cells, which are more likely to lead to inflammation (29–33). Furthermore, it has been suggested that LDL plays a role in the progression of arterial stiffness and that elevated levels of LDL are associated with an elevated risk of ischemic heart disease and coronary artery disease (CAD) (34). After 3 years of follow-up, a prospective study from Copenhagen found that higher LDL levels were associated with a higher risk of ischemic stroke in 38,319 study participants (35).

Stroke has now become one of the chronic non-communicable diseases (NCDs) that seriously endanger the lives and health of the Chinese people, and its disease burden is increasing year by year. However, there are relatively few studies on the association between inflammation and stroke in Chinese residents aged 40 years and older, and even fewer studies on the effect of inflammation combined with dyslipidemia on stroke. In this study, we investigated the effects of inflammation, dyslipidemia (of different types) and their combined effects on stroke, which is conducive to the prevention of stroke. Of course, the study also has the following limitations. First, this population cohort mainly studies dietary changes in China. Compared to the traditional cardiovascular cohort, this may underestimate the number of strokes, leading to skewed results. Second, the questions asked during follow-up showed if someone had a stroke based on what they said themselves and not on a medical diagnosis. In addition, because the information is incomplete, it cannot greatly distinguish between the type of stroke (ischemic stroke and hemorrhagic stroke). Third, the study was conducted in non-consecutive years (2009, 2015 and 2018) and only with a 3-day 24-h dietary recall, household food weighing method, and food frequency questionnaire which may limit the results (inclusively alter them).

In conclusion, the findings of our study demonstrate that the co-occurrence of elevated CRP levels and dyslipidemia may heighten the susceptibility to stroke in middle-aged and older adult individuals in China. Implementing interventions targeting these risk factors may be beneficial for stroke prevention. Consequently, further research is warranted to assess the predictive capacity of elevated CRP levels and dyslipidemia for stroke risk over an extended period of observation.

## Data Availability

The raw data supporting the conclusions of this article will be made available by the authors, without undue reservation.

## References

[1] Saini V Guada L Yavagal DR. Global epidemiology of stroke and access to acute ischemic stroke interventions. Neurology. (2021) 97:S6–S16. 10.1212/WNL.0000000000012781 34785599

[2] Ma Q Li R Wang L Yin P Wang Y Yan C. Temporal trend and attributable risk factors of stroke burden in China, 1990-2019: an analysis for the Global Burden of Disease Study 2019. Lancet Public Health. (2021) 6:e897–906. 10.1016/S2468-2667(21)00228-0 34838196 PMC9047702

[3] Tu W-J Hua Y Yan F Bian H Yang Y Lou M. Prevalence of stroke in China, 2013-2019: a population-based study. Lancet Reg Health West Pac. (2022) 28:100550. 10.1016/j.lanwpc.2022.100550 36507089 PMC9727498

[4] Zhou Y Han W Gong D Man C Fan Y. Hs-CRP in stroke: a meta-analysis. Clin Chim Acta. (2016) 453:21–7. 10.1016/j.cca.2015.11.027 26633855

[5] Lee S Song IU Na SH Jeong DS Chung SW. Association between long-term functional outcome and change in hs-CRP level in patients with acute ischemic stroke. Neurologist. (2020) 25:122–5. 10.1097/NRL.0000000000000278 32925482

[6] Fonseca FA Izar MC. High-sensitivity c-reactive protein and cardiovascular disease across countries and ethnicities. Clinics. (2016) 71:235–42. 10.6061/clinics/2016(04)11 27166776 PMC4825196

[7] Popkin BM Du S Zhai F Zhang B. Cohort Profile: The China Health and Nutrition Survey–monitoring and understanding socio-economic and health change in China, 1989-2011. Int J Epidemiol. (2010) 39:1435–40. 10.1093/ije/dyp322 19887509 PMC2992625

[8] Zhang B Zhai FY Du SF Popkin BM. The China health and nutrition survey, 1989-2011. Obes Rev. (2014) 15:2–7. 10.1111/obr.12119 24341753 PMC3869031

[9] Chen X Liu S Chu J Hu W Sun N Shen Y. Joint effect of elevated-c-reactive protein level and hypertension on new-onset stroke: a nationwide prospective cohort study of CHARLS. Front Public Health. (2022) 10:919506. 10.3389/fpubh.2022.919506 36262245 PMC9573958

[10] Cooney MT Bruckert E Cordero A Corsini A Giannuzzi P. 2016 ESC/EAS guidelines for the management of dyslipidaemias. Eur Heart J. (2016) 37:2999–3058. 10.1093/eurheartj/ehw415 27567407

[11] Yan S Li J Li S Zhang B Du S Gordon-Larsen P. The expanding burden of cardiometabolic risk in China: the China Health and Nutrition Survey. Obes Rev. (2012) 13:810–821. 10.1111/j.1467-789X.2012.01016.x 22738663 PMC3429648

[12] Liu Y Wang J Zhang L Wang C Wu J Zhou Y. Relationship between C-reactive protein and stroke: a large prospective community based study. PLoS ONE. (2014) 9:e107017. 10.1371/journal.pone.0107017 25191699 PMC4156395

[13] van Wijk DF Boekholdt SM Wareham NJ Ahmadi-Abhari S Kastelein JJ Stroes ES. C-reactive protein, fatal and nonfatal coronary artery disease, stroke, and peripheral artery disease in the prospective EPIC-Norfolk cohort study. Arterioscler Thromb Vasc Biol. (2013) 33:2888–94. 10.1161/ATVBAHA.113.301736 24072695

[14] Koenig W Löwel H Baumert J Meisinger C. C-reactive protein modulates risk prediction based on the Framingham Score: implications for future risk assessment: results from a large cohort study in southern Germany. Circulation. (2004) 109:1349–53. 10.1161/01.CIR.0000120707.98922.E3 15023871

[15] Ridker PM Cushman M Stampfer MJ Tracy RP Hennekens CH. Inflammation, aspirin, and the risk of cardiovascular disease in apparently healthy men. N Engl J Med. (1997) 336:973–9. 10.1056/NEJM199704033361401 9077376

[16] Skoblow HF Proulx CM. C-reactive protein, subjective aging, and incident cardiovascular disease: a mediation model. J Gerontol B Psychol Sci Soc Sci. (2022) 77:1654–8. 10.1093/geronb/gbac051 35279030 PMC9434473

[17] Boekholdt SM Hack CE Sandhu MS Luben R Bingham SA Wareham NJ. C-reactive protein levels and coronary artery disease incidence and mortality in apparently healthy men and women: the EPIC-Norfolk prospective population study 1993-2003. Atherosclerosis. (2006) 187:415–22. 10.1016/j.atherosclerosis.2005.09.023 16257408

[18] Deplanque D Amarenco P. Blood lipids and stroke: what more can we do besides reducing low-density lipoprotein cholesterol? Curr Atheroscler Rep. (2011) 13:306–13. 10.1007/s11883-011-0186-z 21706190

[19] Tziomalos K Athyros VG Karagiannis A Mikhailidis DP. Dyslipidemia as a risk factor for ischemic stroke. Curr Top Med Chem. (2009) 9:1291–7. 10.2174/156802609789869628 19849661

[20] Suwanwela NC. Stroke epidemiology in Thailand. J Stroke. (2014) 16:1–7. 10.5853/jos.2014.16.1.1 24741559 PMC3961816

[21] Ohya Y Matsuo R Sato N Irie F Nakamura K Wakisaka Y. Causes of ischemic stroke in young adults versus non-young adults: a multicenter hospital-based observational study. PLoS ONE. (2022) 17:e268481. 10.1371/journal.pone.0268481 35830430 PMC9278748

[22] Yi X Luo H Zhou J Yu M Chen X Tan L. Prevalence of stroke and stroke related risk factors: a population based cross sectional survey in southwestern China. BMC Neurol. (2020) 20:5. 10.1186/s12883-019-1592-z 31910820 PMC6947997

[23] Opoku S Gan Y Fu W Chen D Addo-Yobo E Trofimovitch D. Prevalence and risk factors for dyslipidemia among adults in rural and urban China: findings from the China National Stroke Screening and prevention project (CNSSPP). BMC Public Health. (2019) 19:1500. 10.1186/s12889-019-7827-5 31711454 PMC6849283

[24] Lechner K McKenzie AL Kränkel N Von Schacky C Worm N Nixdorff U. High-risk atherosclerosis and metabolic phenotype: the roles of ectopic adiposity, atherogenic dyslipidemia, and inflammation. Metab Syndr Relat Disord. (2020) 18:176–85. 10.1089/met.2019.0115 32119801 PMC7196362

[25] Hasheminasabgorji E Jha JC. Dyslipidemia, diabetes and atherosclerosis: role of inflammation and ROS-redox-sensitive factors. Biomedicines. (2021) 9:1602. 10.3390/biomedicines9111602 34829831 PMC8615779

[26] Tavridou A Manolopoulos VG. Novel molecules targeting dyslipidemia and atherosclerosis. Curr Med Chem. (2008) 15:792–802. 10.2174/092986708783955482 18393849

[27] Škrha JJ. Diabetes, lipids, and CV risk. Curr Atheroscler Rep. (2021) 23:8. 10.1007/s11883-021-00905-8 33464402

[28] Groenen AG Halmos B Tall AR Westerterp M. Cholesterol efflux pathways, inflammation, and atherosclerosis. Crit Rev Biochem Mol Biol. (2021) 56:426–39. 10.1080/10409238.2021.1925217 34182846 PMC9007272

[29] Poznyak A Grechko AV Poggio P Myasoedova VA Alfieri V Orekhov AN. The diabetes mellitus-atherosclerosis connection: the role of lipid and glucose metabolism and chronic inflammation. Int J Mol Sci. (2020) 21:1835. 10.3390/ijms21051835 32155866 PMC7084712

[30] Lin P Ji HH Li YJ Guo SD. Macrophage plasticity and atherosclerosis therapy. Front Mol Biosci. (2021) 8:679797. 10.3389/fmolb.2021.679797 34026849 PMC8138136

[31] Shapouri-Moghaddam A Mohammadian S Vazini H Taghadosi M Esmaeili SA Mardani F. Macrophage plasticity, polarization, and function in health and disease. J Cell Physiol. (2018) 233:6425–40. 10.1002/jcp.26429 29319160 PMC13482560

[32] Higashi Y. Endothelial function in dyslipidemia: roles of LDL-cholesterol, HDL-cholesterol and triglycerides. Cells. (2023) 12:1293. 10.3390/cells12091293 37174693 PMC10177132

[33] Zysset D Weber B Rihs S Brasseit J Freigang S Riether C. TREM-1 links dyslipidemia to inflammation and lipid deposition in atherosclerosis. Nat Commun. (2016) 7:13151. 10.1038/ncomms13151 27762264 PMC5080444

[34] Liu H Li J Liu F Huang K Cao J Chen S. Efficacy and safety of low levels of low-density lipoprotein cholesterol: trans-ancestry linear and non-linear Mendelian randomization analyses. Eur J Prev Cardiol. (2023) 30:1207–15. 10.1093/eurjpc/zwad111 37040432

[35] Balling M Nordestgaard BG Varbo A Langsted A Kamstrup PR Afzal S. Small dense low-density lipoprotein cholesterol and ischemic stroke. Ann Neurol. (2023) 93:952–64. 10.1002/ana.26598 36606557

