# Supplementary material for: Combined impact of elevated C-reactive protein levels and dyslipidemia on stroke: a CHNS prospective cohort study
Source: Front Public Health. 2024 Aug 23;12:1435004. doi: 10.3389/fpubh.2024.1435004 (PMC11377318; doi:10.3389/fpubh.2024.1435004)
Supplement: Supplementary file 1 [file Table_1.DOCX]

**Table 3.** Odd ratios (OR) and 95% confidence intervals (95% CI) of stroke according to dyslipidemia.

|  | Dyslipidemia | |
| --- | --- | --- |
|  | No | Yes |
| Total |  |  |
| Number of participants | 10193 | 5853 |
| Number of cases | 100 | 74 |
| OR (95%CI) |  |  |
| Model 1 | 1 | 1.45 (1.07, 1.96) |
| Model 2 | 1 | 1.26 (0.91, 1.73) |
| Model 3 | 1 | 1.26 (0.91, 1.74) |
| Model 4 | 1 | 1.06 (0.77, 1.45) |
| Males |  |  |
| Number of participants | 4419 | 2771 |
| Number of cases | 64 | 44 |
| OR (95%CI) |  |  |
| Model 1 | 1 | 1.38 (0.93, 2.05) |
| Model 2 | 1 | 1.23 (0.81, 1.88) |
| Model 3 | 1 | 1.24 (0.82, 1.89) |
| Model 4 | 1 | 1.05 (0.69, 1.59) |
| Females |  |  |
| Number of participants | 5774 | 3082 |
| Number of cases | 36 | 30 |
| OR (95%CI) |  |  |
| Model 1 | 1 | 1.49 (0.93, 2.39) |
| Model 2 | 1 | 1.26 (0.78, 2.06) |
| Model 3 | 1 | 1.28 (0.79, 2.10) |
| Model 4 | 1 | 1.07 (0.66, 1.74) |

Model1: age, sex, education level, geographic region, marital status, and average household income.
Model2: model1 with smoking, drinking, BMI, and physical activity.

Model3: model2 with dietary fat, cholesterol, vegetables, energy, sodium, and red meat.

Model4: model3 with hypertension, diabetes, HS-CRP, and tumor.

**Table 4.** Odd ratios (OR) and 95% confidence intervals (95% CI) of stroke according to different types of dyslipidemia

|  | No | Yes | Type of dyslipidemia | | | |
| --- | --- | --- | --- | --- | --- | --- |
|  |  |  | High Cholesterol | High Triglycerides | High LDL Cholesterol | Low HDL Cholesterol |
| Total |  |  |  |  |  |  |
| Number of participants | 10193 | 5853 | 106 | 1456 | 2051 | 2240 |
| Number of cases | 100 | 74 | 1 | 9 | 34 | 30 |
| OR (95%CI) |  |  |  |  |  |  |
| Model 1 | 1 | 1.45 (1.07, 1.96) | 0.86 (0.12, 6.44) | 0.82 (0.41, 1.63) | 1.84 (1.22, 2.77) | 1.46 (0.95, 2.23) |
| Model 2 | 1 | 1.26 (0.91, 1.73) | 0.84 (0.11, 6.13) | 0.75 (0.37, 1.51) | 1.69 (1.10, 2.58) | 1.17 (0.75, 1.80) |
| Model 3 | 1 | 1.26 (0.91, 1.74) | 0.81 (0.12, 5.60) | 0.75 (0.37, 1.52) | 1.73 (1.13, 2.64) | 1.15 (0.74, 1.78) |
| Model 4 | 1 | 1.06 (0.77, 1.45) | 0.73 (0.09, 5.98) | 0.66 (0.32, 1.35) | 1.56 (1.03, 2.37) | 0.89 (0.58, 1.37) |
| Males |  |  |  |  |  |  |
| Number of participants | 4419 | 2771 | 35 | 637 | 764 | 1335 |
| Number of cases | 64 | 44 | 1 | 5 | 21 | 17 |
| OR (95%CI) |  |  |  |  |  |  |
| Model 1 | 1 | 1.38 (0.93, 2.05) | 1.72 (0.21, 14.06) | 0.83 (0.33, 2.08) | 2.20 (1.30, 3.73) | 1.08 (0.62, 1.88) |
| Model 2 | 1 | 1.23 (0.81, 1.88) | 1.65 (0.21, 12.98) | 0.80 (0.31, 2.05) | 2.08 (1.20, 3.62) | 0.89 (0.50, 1.56) |
| Model 3 | 1 | 1.24 (0.82, 1.89) | 1.53 (0.22, 10.81) | 0.83 (0.32, 2.16) | 2.13 (1.22, 3.71) | 0.88 (0.50, 1.56) |
| Model 4 | 1 | 1.05 (0.69, 1.59) | 1.12 (0.16, 10.88) | 0.72 (0.27, 1.93) | 1.93 (1.12, 3.33) | 0.71 (0.41, 1.26) |
| Females |  |  |  |  |  |  |
| Number of participants | 5774 | 3082 | 71 | 819 | 1287 | 905 |
| Number of cases | 36 | 30 | 0 | 4 | 13 | 13 |
| OR (95%CI) |  |  |  |  |  |  |
| Model 1 | 1 | 1.49 (0.93, 2.39) | — | 0.79 (0.28, 2.26) | 1.43 (0.76, 2.68) | 2.41 (1.28, 4.54) |
| Model 2 | 1 | 1.26 (0.78, 2.06) | — | 0.67 (0.23, 1.91) | 1.27 (0.67, 2.40) | 1.91 (0.99, 3.68) |
| Model 3 | 1 | 1.28 (0.79, 2.10) | — | 0.67 (0.23, 1.94) | 1.33 (0.70, 2.53) | 1.89 (0.97, 3.68) |
| Model 4 | 1 | 1.07 (0.66, 1.74) | — | 0.58 (0.22, 1.68) | 1.14 (0.60, 2.19) | 1.44 (0.74, 2.80 ) |

Model1: age, sex, education level, geographic region, marital status, and average household income.
Model2: model1 with smoking, drinking, BMI, and physical activity.

Model3: model2 with dietary fat, cholesterol, vegetables, energy, sodium, and red meat.

Model4: model3 with hypertension, diabetes, and tumor.
